# Short-term renal and cardiac outcomes of combined ARNI and SGLT2 therapy in AHF patients: a prospective single-arm interventional study

**DOI:** 10.3389/fcvm.2026.1815471

**Published:** 2026-05-01

**Authors:** MingLei Zhang, ZiYing Huang, ChenChen Wang, YanDan Xu

**Affiliations:** 1 Department of Pharmacy, Quzhou KeCheng People’s Hospital, Quzhou, Zhejiang, China; 2 Department of Traditional Chinese Medicine and Science Education, Quzhou KeCheng People’s Hospital, Quzhou, Zhejiang, China

**Keywords:** acute heart failure, ARNI, cardiovascular biomarkers, renal function, SGLT2, urine volume

## Abstract

Acute heart failure (AHF) requires immediate intervention, with diuretics central to managing volume overload. We conducted a prospective single-arm study to explore changes in diuretic and renal outcomes following initiation of combined angiotensin receptor neprilysin inhibitor (ARNI) and sodium-glucose cotransporter 2 (SGLT2) inhibitors therapy in AHF patients. We enrolled 120 patients with left ventricular ejection fraction ≤40% and signs of fluid overload, initiation of combined ARNI and SGLT2 inhibitor therapy post-hemodynamic stabilization. Urine volume, renal function, and cardiovascular biomarkers were assessed over 9 days. Urine volume and diuretic response increased significantly on day 1, stabilizing above baseline levels. Cardiovascular biomarkers showed improvements, with decreased NT-proBNP and increased LVEF, without affecting eGFR. Increased diuresis and improved cardiac function parameters were observed during combination therapy. These hypothesis-generating findings support the need for randomized controlled trials to evaluate the clinical utility of this combination in AHF management. The short-term nature of the study and lack of a control group are limitations, which preclude definitive causal attribution of the observed effects. This study has been registered with the Chinese Clinical Trial Registry under registration number ChiCTR2600118845.

**Clinical Trial Registration:**

Chinese Clinical Trial Registry (ChiCTR2600118845).

## Introduction

Acute heart failure (AHF) constitutes an acute clinical syndrome precipitated by diverse etiologies, marked by the rapid onset or acute exacerbation of heart failure symptoms and signs, accompanied by elevated plasma natriuretic peptide levels. This condition is frequently life-threatening and demands prompt medical intervention, often requiring emergency hospitalization. The prognosis for acute heart failure remains poor, with an in-hospital mortality rate of 12%, a readmission rate of approximately 45% within one year, and a one-year mortality rate of around 22% (1).

The management of acute heart failure constitutes a complex challenge, primarily due to the intricate task of maintaining hemodynamic stability while effectively managing volume status (2). In cases where patients with heart failure are hospitalized due to acute decompensation, the administration of diuretics to address volume overload is a fundamental component of treatment. This approach aims to alleviate congestion-related organ damage and improve patient prognosis.

The angiotensin receptor neprilysin inhibitor (ARNI), exemplified by sacubitril/valsartan, has emerged as a promising therapeutic strategy for heart failure management. The PARADIGM-HF trial demonstrated that ARNI significantly reduced the risk of cardiovascular mortality and heart failure-related hospitalizations compared to the ACE inhibitor enalapril, signifying a pivotal shift in the treatment paradigm (3). Subsequent investigations, including the PIONEER-HF trial, have shown significant reductions in NT-proBNP levels and rehospitalization rates, further substantiating the advantages of early ARNI initiation in patients recently hospitalized for AHF, thereby underscoring its potential utility in acute clinical settings (4).

Sodium-glucose cotransporter 2 (SGLT2) inhibitors were originally developed for the management of diabetes and have subsequently emerged as a promising class of diuretics for volume management in heart failure. Their distinctive mechanism of action in the renal tubules facilitates both natriuresis and osmotic diuresis, thereby promoting effective fluid removal and alleviation of congestion (5). In addition to their diuretic properties, SGLT2 inhibitors confer cardiovascular benefits and potential renal advantages, such as reducing cardiovascular mortality, preserving glomerular filtration rate (GFR), decreasing proteinuria, and lowering rates of rehospitalization (6).

ARNI and SGLT2 inhibitors are currently regarded as the primary therapeutic strategy for managing chronic heart failure, providing a synergistic mechanism to mitigate morbidity and mortality. Nonetheless, the utilization of this combination in the context of acute heart failure has not been thoroughly investigated. This study seeks to examine the diuretic and renal effects of ARNI in conjunction with SGLT2 inhibitors in patients hospitalized with AHF, with a particular emphasis on the impact of early treatment on systemic congestion and safety.

## Methods

### Patient recruitment

Individuals aged ≥18 years with a preliminary diagnosis of acute decompensated heart failure requiring hospitalization were eligible. All patients had a left ventricular ejection fraction (LVEF) ≤ 40% (heart failure with reduced ejection fraction, HFrEF) and signs of fluid overload, defined by the presence of at least two of the following: peripheral edema (≥1+), jugular venous distension, pulmonary congestion on chest radiography, or orthopnea. Biochemical confirmation of heart failure required NT-proBNP ≥ 1,600 pg/mL (primary assay used at our institution). Enrollment occurred within 24 h to 10 days following initial admission. Participants must demonstrate hemodynamic stability, defined as maintaining a systolic blood pressure of at least 100 mmHg for the preceding 6 h, without an increase in intravenous diuretic dosage, and without the use of intravenous vasodilators or inotropes in the previous 24 h. Heart rate < 100 bpm and urine output >0.5 mL/kg/h without clinical signs of hypoperfusion. These criteria were assessed by the treating cardiologist and verified by the study investigators prior to treatment initiation. Written informed consent was obtained from all participants in this study. Other exclusion criteria include: (1) Cardiogenic shock, severe respiratory distress with significant hypoxemia, hematologic diseases, autoimmune disorders, severe hepatic or renal disease (eGFR ≤ 30 mL/min/1.73 m^2^), severe arrhythmias, myocarditis, acute myocardial infarction, severe electrolyte disturbances, acid-base imbalances, severe infections, or malignancies; (2) Contraindications to the use of sacubitril/valsartan or dapagliflozin; (3) Mental health disorders or communication barriers; (4) History of drug allergies; (5) Participation in other clinical trials within the last two months. Patients were classified as having either *de novo* acute heart failure (first presentation without prior heart failure diagnosis) or acute decompensation of chronic heart failure (worsening of previously diagnosed chronic heart failure). Underlying heart failure etiologies were determined based on medical records, coronary angiography, echocardiography, and clinical history, and were categorized as: ischemic cardiomyopathy, dilated cardiomyopathy, hypertensive heart disease, or valvular heart disease. Pre-admission guideline-directed medical therapy (GDMT) was recorded, including prior use of ACEis, ARBs, ARNI, β-blockers, MRAs, and SGLT2 inhibitors. This was a prospective, single-arm interventional study designed to evaluate changes in clinical parameters following initiation of combined ARNI and SGLT2 inhibitor therapy. No concurrent control group was included. This study was approved by the Ethics Committee of Kecheng People's Hospital, Quzhou City, under the acceptance number KCYY2023-K04-02.

### Study timeline and treatment protocol

The study comprised a 2-day baseline period followed by 9 days of combination therapy. Key steps are as follows:

- Day −2 (48–24 h before treatment initiation): Baseline 24 h urine collection started. Patients continued their pre-admission ACEi or ARB therapy.
- Day −1 (24–0 h before treatment): Baseline urine collection completed. For patients on ACEi, a 36 h washout was initiated (last ACEi dose on Day −2 evening). Patients on ARB continued their usual dose.
- Day 0 (treatment initiation, 7 AM): ACEi or ARB discontinued (36 h washout for ACEi; ARB stopped without washout). First dose of ARNI (sacubitril/valsartan 24 mg/26 mg) administered orally twice daily, and dapagliflozin 10 mg administered orally once daily. A new 24 h urine collection started immediately after the first dose.
- Day 1 to Day 9: Combination therapy continued at the same doses. Urine collections were performed on Days 1, 3, 5, 7, and 9 (each from 7 AM to 7 AM the following day).

The selection of biomarkers is informed by the sub-study of PIONEER-HF (7), which includes the high-sensitivity measurement of cardiac troponin（hs-TnT）, Soluble ST2 (sST2), urinary cGMP (ucGMP), NT-proBNP and eGFR (calculated using the CKD-EPI equation) are measured on Days −2 and 14 (8). Furthermore,parameters such as diuretic response (DR), systolic blood pressure (SBP), body weight (BW), hematocrit (HCT), plasma volume (PV), and urine albumin to creatinine ratio are documented.The diuretic response is calculated as UV divided by the intravenous furosemide dose in 40 mg equivalents. The diuretic dosage conversion is as follows:1 mg of Bumetanide is equivalent to 20 mg of Torsemide, 40 mg of intravenous Furosemide, or 80 mg oral Furosemide (9). Plasma volume is calculated using the formula (1—hematocrit) × [a + (b × weight in kg)], where a = 1,530 for males and 864 for females, and b = 41 for males and 47.9 for females (10).

### Background and concomitant therapies

Prior to study therapy initiation, all patients received intravenous furosemide for management of volume overload. The baseline daily furosemide dose (in 40 mg equivalents) was recorded for the 24 h period immediately preceding treatment initiation (Day −2 to Day −1). The median baseline furosemide dose was 80 mg/day [IQR: 60–120 mg/day]. This dose was used as a reference for calculating diuretic response during the study period. As part of the hemodynamic stabilization criteria (see above), patients were required to have no use of intravenous vasodilators (e.g., nitroglycerin, nitroprusside) or inotropic agents (e.g., dobutamine, milrinone) for at least 24 h prior to study therapy initiation. During the 9-day study period after treatment initiation, no patients received intravenous vasodilators or inotropes, as this would have reflected hemodynamic instability and precluded continued participation. All patients received IV furosemide starting at 40 mg once or twice daily, titrated according to urine output, body weight, and clinical congestion. The total daily furosemide dose was recorded and used to calculate diuretic response (urine output per 40 mg furosemide equivalent). GDMT: Background β-blockers (bisoprolol, carvedilol or metoprolol succinate), mineralocorticoid receptor antagonists (spironolactone or eplerenone), and digoxin were continued at stable pre-admission doses. No dose adjustments were made during the 9-day study period unless clinically required for safety.

### Outcome measures

The primary outcome was the change in 24-hour urine volume (UV) from baseline (Day −2) to Day 1 and over the 9-day observation period (Days −2, 0, 1, 3, 5, 7, and 9). Secondary outcomes included: DR, reflects diuretic efficiency and has prognostic value in heart failure (11). Changes in cardiovascular biomarkers: NT-proBNP, sST2, hs-TnT, ucGMP, and LVEF. Safety outcomes: incidence of acute kidney injury (AKI) defined by KDIGO criteria (increase in serum creatinine ≥0.3 mg/dL within 48 h or ≥1.5 times baseline), symptomatic hypotension, and clinically significant electrolyte disturbances (serum potassium <3.5 or >5.5 mmol/L, or sodium <130 mmol/L).

### Statistical analysis

Normality of the distribution for each continuous variable was assessed using the Shapiro–Wilk test. For comparisons between two time points (e.g., baseline vs. Day 14), normally distributed variables were analyzed using the paired t-test. For variables that were not normally distributed (as determined by the Shapiro–Wilk test, *P* < 0.05), longitudinal comparisons across multiple time points were performed using the Friedman test (non-parametric repeated measures). When the overall Friedman test was significant, *post-hoc* pairwise comparisons against baseline were conducted using Dunn's test with Bonferroni adjustment. For two-timepoint comparisons of non-normally distributed variables, the Wilcoxon signed-rank test was used. Results are presented as mean ± SD for normally distributed variables and as median (IQR, IQR) for non-normally distributed variables. For repeated measurements of UV and DR collected at seven time points (Day −2, 0, 1, 3, 5, 7, and 9), a repeated measures ANOVA with Greenhouse-Geisser correction was applied to account for sphericity violations. When the overall time effect was significant, *post-hoc* comparisons against baseline were performed using Bonferroni adjustment. To explore baseline factors associated with UV on Day 1, multiple linear regression was performed with entry of prespecified variables (age, BMI, SBP, PV, HCT, NT-proBNP, LVEF, and oral furosemide dose). No adjustment for time-varying confounders (e.g., daily loop diuretic dose) was undertaken due to the exploratory nature of this single-arm study. All analyses were performed using SPSS version 20.0. A two-tailed *P* < 0.05 was considered statistically significant.

## Results

### Study population

A total of 120 patients were enrolled in this study. Baseline characteristics are detailed in Table 1. The median age was 60 years [IQR: 49, 70], and 21.6% were female. The median BMI was 29.8 kg/m^2^ [IQR: 24.3, 35.7]. Mean systolic blood pressure was 117 ± 10 mmHg, and mean heart rate was 79 ± 17 bpm. Mean eGFR was 61 ± 12 mL/min/1.73 m², and median LVEF was 26% [IQR: 18, 30]. Median NT-proBNP was 2,760 pg/mL [IQR: 2,420, 3,088].

**Table 1:** Baseline characteristics among patients.

Variables	120
Age, median [IQR], years	60[49, 70]
Female sex, no. (%)	26 (21.6)
BMI, median ± SD, kg/m^2^	29.8 ± 4.3
Heart failure characteristics	
*De novo* acute heart failure, *n* (%)	22 (18.3)
Acute decompensation of chronic HF, *n* (%)	98 (81.7)
Underlying etiology	
Ischemic cardiomyopathy, *n* (%)	58 (48.3)
Dilated cardiomyopathy, *n* (%)	42 (35.0)
Hypertensive heart disease, *n* (%)	14 (11.7)
Valvular heart disease, *n* (%)	6 (5.0)
Pre-admission GDMT	
Baseline IV furosemide dose, median [IQR], mg/day	80 [60, 120]
ACEi, *n* (%)	60 (50.0)
ARB, *n* (%)	65 (54.2)
ARNI, *n* (%)	12 (10.0)
β*-*blocker, *n* (%)	37 (30.8)
MRA, *n* (%)	32 (26.7)
SGLT2 inhibitor, *n* (%)	28 (23.3)
Any GDMT, *n* (%)	85 (70.8)
Dual GDMT (RAASi + β-blocker), *n* (%)	41 (34.2)
Vital sings and laboratory data at baseline	
SBP, median ± SD, mmHg	117 ± 10
Pulse, median ± SD, b.p.m.	79 ± 17
eGFR, median ± SD, mL/min/1.73 m^2^	61 ± 12
LVEF, median[IQR], %	26[18, 30]
NT-proBNP, median [IQR], pg/mL	2,760[2,420.25, 3,087.5]

Data are presented as mean ± SD for normally distributed variables and as median [IQR] for non-normally distributed variables. Normality was assessed using the Shapiro–Wilk test. ACEi, angiotensin-converting enzyme inhibitor; ARB, angiotensin receptor blocker; ARNI, angiotensin receptor neprilysin inhibitor; BMI, body mass index; eGFR, estimated glomerular filtration rate; GDMT, guideline-directed medical therapy; IQR, interquartile range; LVEF, left ventricular ejection fraction; MRA, mineralocorticoid receptor antagonist; NT-proBNP, N-terminal pro-B-type natriuretic peptide; SBP, systolic blood pressure; SGLT2, sodium-glucose cotransporter 2.

#### Heart failure characteristics

Among the 120 patients, 98 (81.7%) had acute decompensation of chronic heart failure, while 22 (18.3%) presented with *de novo* acute heart failure. Underlying etiologies included ischemic cardiomyopathy in 58 patients (48.3%), dilated cardiomyopathy in 42 (35.0%), hypertensive heart disease in 14 (11.7%), and valvular heart disease in 6 (5.0%).

#### Pre-admission GDMT

Prior to admission, 60 patients (50.0%) were receiving ACEis, 65 (54.2%) were receiving ARBs, and 12 (10.0%) were receiving ARNI. β-blockers were used by 37 patients (30.8%), MRAs by 32 (26.7%), and SGLT2 inhibitors by 28 (23.3%). A total of 85 patients (70.8%) were on at least one GDMT agent, and 41 (34.2%) were on dual GDMT (ACEi/ARB/ARNI plus β-blocker) prior to admission. Additional details regarding medication and medical history are provided in Table 1.

### Urine volume and renal function

**Figure 1:**
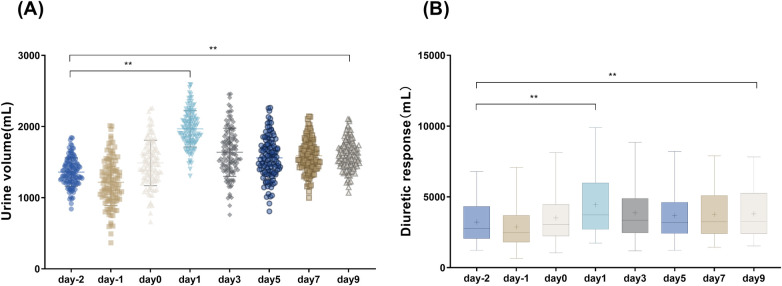
Changes in urine output and diuretic response before and after combined ARNI and SGLT2 inhibitor initiation. Urine volume **(A)** was analyzed using repeated measures ANOVA with Bonferroni post-hoc. Diuretic response **(B)** was analyzed using the Friedman test with Dunn's post-hoc. *P < 0.05, **P < 0.01 compared with baseline (Day −2). ARNI, angiotensin receptor neprilysin inhibitor; SGLT2, sodium-glucose cotransporter 2.

UV, which was normally distributed, is reported as mean ± SD. Compared with baseline (Day −2), UV increased significantly on Day 0 (608 ± 56 mL, *P* < 0.001, paired *t*-test) and remained elevated through Day 9 (245 ± 42 mL at Day 9, *P* = 0.003). DR was not normally distributed (Shapiro–Wilk *P* < 0.05). Median (IQR) values are reported. The Friedman test revealed a significant overall time effect (*P* < 0.001). Compared with baseline [Day −2: median 45 (IQR 32–58) mL per 40 mg furosemide equivalent], DR increased significantly on Day 0 [median 82 (IQR 64–103) mL, *P* < 0.001, Dunn's test] and remained above baseline through Day 9 [median 58 (IQR 44–72) mL, *P* = 0.01]. Detailed longitudinal data are presented in Figure 1. No significant changes in eGFR were observed over the 9-day study period (*P* = 0.32 for repeated measures ANOVA). However, given the short follow-up duration and the known hemodynamic effects of ARNI and SGLT2 inhibitors on eGFR shortly after initiation, this finding should not be interpreted as evidence of long-term renal safety.

### Cardiovascular biomarkers

**Figure 2:**
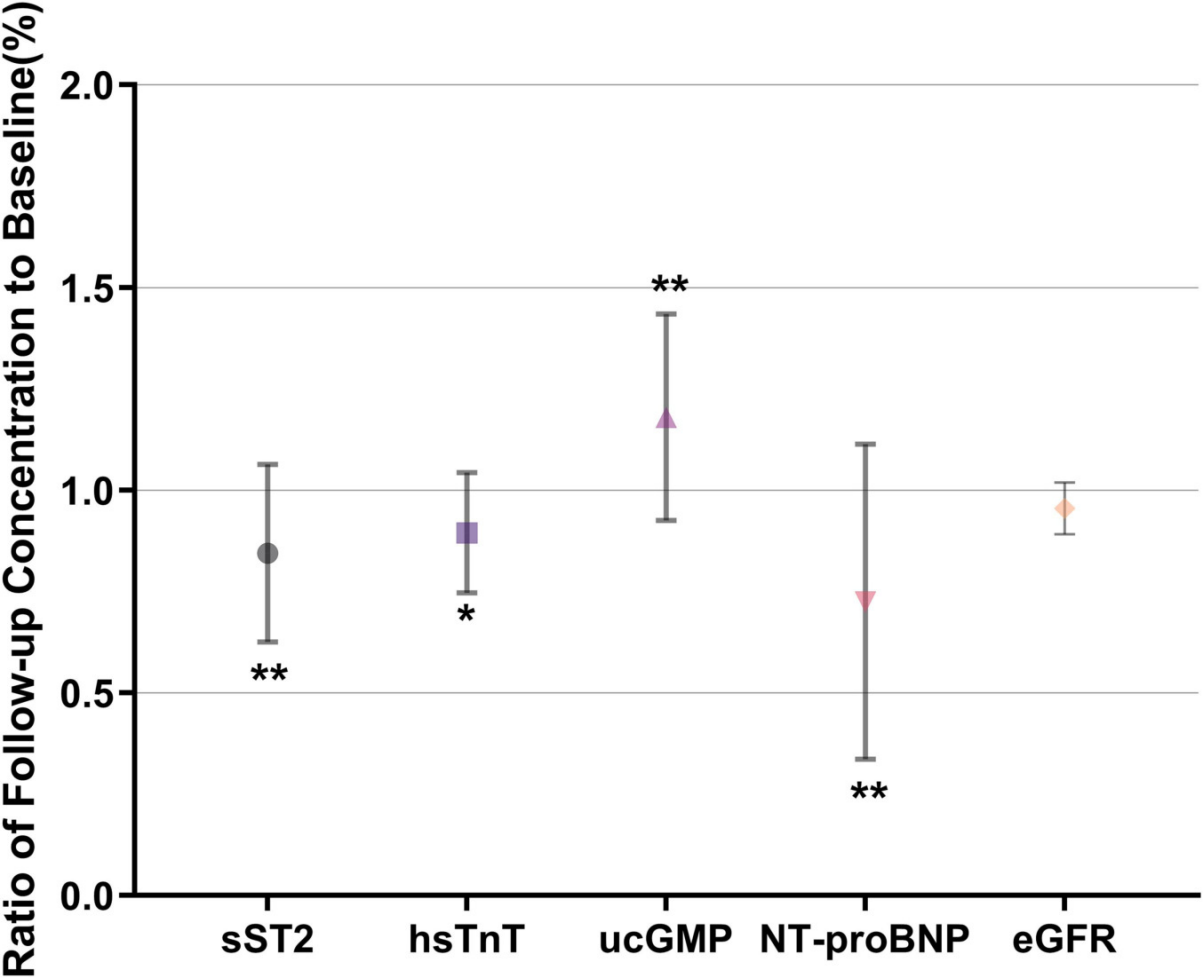
Changes in cardiovascular biomarkers after SGLT2 initiation by ARNI. (*P < 0.05, **P < 0.01) ARNI, angiotensin receptor neprilysin inhibitor; SGLT2, sodium-glucose cotransporter 2.

On Day 14, the initiation of ARNI and SGLT2 therapy resulted in a reduction in SBP, BW, HCT, PV, and NT-proBNP levels (Table 2). Specifically, the sST2 level decreased to 69% of its baseline value, while hs-TnT decreased to 79% of its baseline. In contrast, ucGMP and LVEF levels increased to 136% and 150% of their baseline values, respectively. NT-proBNP levels were reduced to 45% of the baseline (Figure 2). Additionally, an increase in the ucGMP/NT-proBNP ratio was observed. In an exploratory multiple linear regression model (Table 3), higher baseline NT-proBNP (β = 0.5988, *P* < 0.0001) and higher BMI (β = 0.0978, *P* = 0.0362) were independently associated with greater urine output on Day 1. This analysis should be interpreted as hypothesis-generating, as it did not account for time-varying confounders such as daily loop diuretic dose.

**Table 2:** Changes in parameters before and after initiation of ARNI and SGLT2.

Parameters	Baseline (Day −2)	Follow-up (Day 14)	*p*-value*
SBP, mean ± SD, mmHg	129 ± 15.3	112 ± 16.3	<0.001
BW, mean ± SD, kg	64.8 ± 18.5	61.9 ± 19.8	<0.001
HCT, mean ± SD, %	38.6 ± 3.2	42.4 ± 4.1	<0.001
PV, mean ± SD, mL	2,525 ± 325.7	2,289 ± 300.3	<0.001
ucGMP/NT-proBNP ratio, median [IQR]	0.26[0.24–0.29]	1.52[1.02–1.96]	<0.001^†^
LVEF, mean ± SD, %	26.1 ± 4.7	39.1 ± 5.6	<0.001

Paired *t*-test unless otherwise indicated. †Wilcoxon signed-rank test*.

BW, body weight; HCT, hematocrit; LVEF, left ventricular ejection fraction; PV, plasma volume; SBP, systolic blood pressure; ucGMP, urinary cyclic guanosine monophosphate; NT-proBNP, N-terminal pro-B-type natriuretic peptide.

**Table 3:** Multiple linear regression analysis of factors associated with urine output on day0.

Factors	Estimate	Standard error	*t*-value	*p*-value
Age	−0.1161	0.1024	1.133	0.2596
BMI	0.09775	0.04609	2.121	0.0362
SBP	0.1683	0.1042	1.616	0.1090
PV	0.0002277	0.0004405	0.5168	0.6063
HCT	1.673	1.103	1.517	0.1322
NT-proBNP	0.5988	0.03083	19.42	<0.0001
LVEF	−0.09783	0.1050	0.9321	0.3533
Oral furosemide dose	0.0003908	0.004034	0.09687	0.9230

BMI, body mass index; SBP, systolic blood pressure; PV, plasma volume; HCT, hematocrit; NT-proBNP, N-terminal pro b-type natriuretic peptide; LVEF, left ventricular ejection fraction.

### Safety outcomes

During the 9-day treatment period and up to Day 14 follow-up, no cases of AKI were observed according to KDIGO criteria. Serum creatinine remained stable throughout the study period, with no patient experiencing a ≥ 0.3 mg/dL increase within 48 h or a ≥ 1.5-fold increase from baseline. No episodes of symptomatic hypotension requiring dose reduction or discontinuation of study medications occurred. Although systolic blood pressure decreased from baseline (129 ± 15.3 mmHg to 112 ± 16.3 mmHg, *P* < 0.001), all patients remained asymptomatic, and no intervention was necessary. No clinically significant electrolyte disturbances were documented. Serum potassium remained within the normal range (3.5–5.5 mmol/L) for all patients, and no cases of hyperkalemia or hypokalemia requiring treatment were recorded. Serum sodium values were consistently ≥135 mmol/L throughout the study period. No participants withdrew from the study due to adverse drug reactions or other clinical adverse events.

## Discussion

In this study, we documented changes in 24 h urine output and renal function parameters during combined ARNI + SGLT2 inhibitor therapy in AHF inpatients. Urine output and diuretic response increased significantly from baseline on treatment day 1, with values stabilizing and maintaining significantly higher levels than baseline throughout the 9-day observation period.

ARNI facilitate natriuresis and diuresis by inhibiting neprilysin, resulting in increased levels of natriuretic peptides. These peptides enhance renal blood flow and GFR, thereby promoting the excretion of sodium and water (12). In patients with heart failure, this mechanism is particularly advantageous as it aids in alleviating congestion and reducing cardiac preload (13).

SGLT2 inhibitors facilitate osmotic diuresis by inhibiting glucose reabsorption in the proximal tubule, resulting in increased excretion of glucose and sodium (14). This mechanism not only aids in weight reduction and enhanced glycemic control but also diminishes plasma volume, which is essential for the management of heart failure (15). Moreover, research has demonstrated that the initiation of SGLT2 inhibitors significantly reduces NT-proBNP levels, which is associated with an improved diuretic response (16). The concurrent administration of ARNI and SGLT2 inhibitors may synergistically enhance diuretic efficacy by targeting distinct renal physiological pathways. This effect is particularly notable in patients with elevated NT-proBNP levels, thereby potentially improving clinical outcomes in the management of acute heart failure (17). Since the initial publication of the EMPULSE trial (18), accumulating evidence has supported the early initiation of SGLT2 inhibitors in patients hospitalized for acute heart failure. The DAPA-ACT trial demonstrated that dapagliflozin initiated during hospitalization for AHF was well tolerated and associated with improved diuretic response (19). A recent meta-analysis confirmed that early initiation of SGLT2 inhibitors in AHF reduces the risk of worsening heart failure and rehospitalization without increasing the risk of acute kidney injury (20). These findings align with our observation that adding dapagliflozin to ARNI therapy was associated with increased urine output and stable eGFR over 9 days. However, our single-arm design precludes direct comparison, and the combined effect of ARNI plus SGLT2 inhibitor in AHF warrants further investigation in randomized controlled trials.

Furthermore, the administration of ARNI has been linked to beneficial alterations in plasma volume and extracellular fluid volume, potentially augmenting the diuretic response. Empirical evidence indicates that ARNI induces a substantial decrease in plasma volume among heart failure patients, thereby enhancing the effectiveness of SGLT2 inhibitors, as corroborated by the findings of the present study (2,525 ± 325.7 to 2,289 ± 300.3) (21). Moreover, the renal protective properties of ARNI, such as the mitigation of renal fibrosis and inflammation, may improve overall renal function, facilitating a more effective response to diuretics (22).

Following the initiation of ARNI and SGLT2 inhibitors, several biomarkers and clinical parameters exhibited significant alterations. Notably, reductions were observed in SBP, BW, HCT, and PV. Concurrently, levels of NT-proBNP, sST2, and hs-TnT also decreased. In contrast, there was an increase in ucGMP and LVEF, indicative of enhanced cardiac function. During the 9-day observation period, eGFR remained stable with no evidence of acute kidney injury. However, this short-term stability should be interpreted with caution, as both ARNI and SGLT2 inhibitors are known to induce transient hemodynamic reductions in eGFR early after initiation, which do not necessarily reflect structural renal impairment (23, 24). Longer-term follow-up is required to assess sustained renal safety. Within the context of HFrEF, the PARADIGM-HF trial has yielded substantial insights into the prognostic utility of biomarkers, particularly the ucGMP to BNP ratio. This ratio has been demonstrated to correlate with clinical outcomes, indicating that patients with a higher ucGMP/BNP ratio experience more favorable prognoses compared to those with lower ratios. Specifically, the trial revealed that elevated ucGMP/BNP ratios were associated with a diminished risk of adverse outcomes, such as heart failure hospitalization and cardiovascular mortality, highlighting the potential application of this biomarker in clinical practice for risk stratification in HFrEF patients (25). In the present study, an elevated ucGMP/NT-proBNP ratio was also observed.

The observation that both obesity and NT-proBNP levels serve as predictors of enhanced diuresis following the initiation of ARNI and SGLT2 therapy in patients with acute heart failure is of significant academic interest. The interplay between obesity and NT-proBNP levels warrants particular attention. Obese individuals frequently exhibit lower NT-proBNP levels, which can complicate the evaluation of heart failure severity and the response to therapeutic interventions (26). Nevertheless, achieving NT-proBNP levels of ≤1,000 pg/mL has been correlated with improved clinical outcomes, irrespective of obesity status (27). This finding indicates that monitoring NT-proBNP levels may offer critical insights into the efficacy of diuretic therapy in heart failure patients, particularly among those with obesity (28).

The advantageous effects of ARNI and SGLT2 inhibitors on cardiac and renal parameters have been substantiated by numerous studies. Specifically, the concurrent use of ARNI and SGLT2 inhibitors has demonstrated improvements in cardiac function and clinical outcomes in patients with HFrEF, while preserving renal stability (29). Furthermore, NT-proBNP levels are recognized as a critical biomarker for evaluating the severity of heart failure and the response to treatment, with reductions in NT-proBNP being associated with enhanced outcomes (30). The prognostic significance of sST2 and hs-TnT as markers in heart failure has also been highlighted, underscoring their utility in monitoring treatment efficacy (31).

Additionally, the initiation of SGLT2 inhibitors has been linked to beneficial alterations in volume status and blood pressure, contributing to the overall improvement in heart failure management (32). These findings underscore the importance of monitoring these biomarkers and clinical parameters in patients receiving ARNI and SGLT2 inhibitor therapy to optimize therapeutic strategies and improve patient outcomes. Importantly, the favorable diuretic and cardiac effects were observed without evidence of renal impairment, symptomatic hypotension, or electrolyte disturbances, suggesting that combined ARNI and SGLT2 inhibitor therapy may have an acceptable short-term safety profile in hemodynamically stable AHF patients. However, these safety findings require confirmation in larger, controlled studies with longer follow-up.

### Comparison with landmark trials

Our findings are best interpreted in the context of prior randomized trials that evaluated ARNI or SGLT2 inhibitors individually in AHF. The PIONEER-HF trial demonstrated that initiating sacubitril/valsartan in hemodynamically stable AHF patients reduced NT-proBNP more effectively than enalapril over 8 weeks (4). The EMPULSE trial showed that empagliflozin initiated in hospitalized AHF patients improved clinical outcomes (a hierarchical composite of all-cause death, heart failure events, and quality of life) compared with placebo, with benefits seen as early as 15 days (18). Our study extends these observations by reporting the combination of both agents started simultaneously after hemodynamic stabilization. While the direction of effect on biomarkers (reduced NT-proBNP, increased LVEF) is consistent with these trials, our single-arm design prevents direct comparison of effect sizes or attribution of causality. Moreover, unlike PIONEER-HF and EMPULSE, our study did not include a control group, and the follow-up was shorter (9–14 days). Nevertheless, the temporal association we observed-rapid improvement in urine output and diuretic response within 24 h of combination therapy-suggests a potential additive or synergistic diuretic effect that warrants investigation in future randomized trials.

### Methodological considerations and interpretation

Several factors limit the causal interpretation of our findings. First, the absence of a control group means that the observed improvements could be partly or wholly attributable to the natural history of AHF, where patients often experience spontaneous diuresis and clinical improvement after admission, independent of the studied intervention. Second, all patients received background loop diuretics (IV furosemide), which were titrated clinically. Although we accounted for loop diuretic dose by calculating diuretic response, we cannot exclude the possibility that changes in furosemide dosing (e.g., dose reduction after initial response) influenced subsequent urine output. Third, regression to the mean may have contributed to the observed decline in NT-proBNP and increase in LVEF, as patients were enrolled during an acute decompensation with extreme values. Fourth, the short follow-up (9 days for urine output, 14 days for biomarkers) does not address whether the observed effects are sustained or translate into improved hard outcomes such as mortality or rehospitalization. Given these limitations, our findings should be considered hypothesis-generating rather than confirmatory. They provide preliminary evidence that combined ARNI and SGLT2 inhibitor therapy is feasible and associated with favorable short-term changes in diuretic and cardiac parameters, without obvious safety signals in this selected, hemodynamically stable population. Future randomized, placebo-controlled trials with longer follow-up are necessary to establish efficacy and safety.

## Conclusion

In this prospective single-arm study of hemodynamically stable AHF patients, initiation of combined ARNI and SGLT2 inhibitor therapy was associated with increased urine output, improved diuretic response, and favorable changes in cardiac biomarkers over 9–14 days, without short-term renal safety concerns. These hypothesis-generating findings suggest that the combination may offer additive diuretic and cardiac benefits beyond standard care. However, due to the lack of a control group and other methodological limitations, causality cannot be inferred. Randomized controlled trials are needed to determine whether this combination strategy improves clinically meaningful outcomes in AHF.

## Limitations

This study has several methodological limitations that should be considered when interpreting the findings.

First, the study employed a single-arm design without a concurrent control group. As such, the observed changes in urine output, biomarkers, and clinical parameters cannot be definitively attributed to the intervention. Potential confounding factors include: Natural disease trajectory: Patients hospitalized with AHF often experience spontaneous clinical improvement in congestion and hemodynamic status during the early phase of hospitalization, independent of any specific intervention. Background therapies: All patients continued to receive standard care, including intravenous loop diuretics, *β*-blockers, and MRAs, which may have contributed to the observed effects and cannot be separated from the effects of the studied combination. Regression to the mean: Patients were enrolled during an acute decompensation episode; extreme baseline values (e.g., high NT-proBNP, low urine output) may show improvement toward the mean irrespective of treatment.

Second, the short-term follow-up (9 days for urine output and diuretic response, 14 days for certain biomarkers) limits our ability to assess the durability of the observed effects or to draw conclusions about long-term clinical outcomes such as mortality, rehospitalization, or sustained improvement in cardiac function.

Third, several statistical limitations should be noted. Although we applied repeated measures ANOVA for normally distributed variables and the Friedman test for non-normally distributed variables, the longitudinal analyses did not include time-varying covariates (e.g., daily loop diuretic dose) that may have influenced diuretic response. Additionally, the multiple linear regression model was exploratory and did not adjust for potential confounders beyond baseline variables. Given the single-arm design, the observed associations do not imply causality.

Fourth, the sample size, while adequate for detecting the primary outcome, was not powered to detect rare adverse events. Although no acute kidney injury, symptomatic hypotension, or clinically significant electrolyte disturbances were documented, larger randomized studies are needed to fully characterize the safety profile of this combination in AHF.

Fifth, the assessment of renal safety is limited by the short follow-up duration (9 days). Both ARNI and SGLT2 inhibitors can cause early, transient declines in eGFR due to hemodynamic effects; such changes may not be captured within the first 9 days, and longer-term renal outcomes (e.g., sustained eGFR decline, progression to chronic kidney disease) cannot be evaluated from this study. Therefore, our finding of stable eGFR should be interpreted as indicating the absence of acute kidney injury during the initial treatment phase, rather than as proof of long-term renal safety.

Finally, this was a hypothesis-generating study conducted at a single center. The findings require confirmation in multicenter, randomized, placebo-controlled trials with longer follow-up before any conclusions regarding efficacy or safety can be drawn for clinical practice.

## Data Availability

The original contributions presented in the study are included in the article/Supplementary Material, further inquiries can be directed to the corresponding author/s.
